# Farnesoid X receptor and bile acids regulate vitamin A storage

**DOI:** 10.1038/s41598-019-55988-w

**Published:** 2019-12-20

**Authors:** Ali Saeed, Jing Yang, Janette Heegsma, Albert K. Groen, Saskia W. C. van Mil, Coen C. Paulusma, Lu Zhou, Bangmao Wang, Klaas Nico Faber

**Affiliations:** 1 Department of Gastroenterology and Hepatology, University of Groningen, Groningen, The Netherlands; 2 Laboratory Medicine, University of Groningen, Groningen, The Netherlands; 3 Department of Pediatrics, Center for Liver, Digestive, and Metabolic Diseases, University Medical Center Groningen, University of Groningen, Groningen, The Netherlands; 4 Amsterdam UMC, University of Amsterdam, Tytgat Institute for Liver and Intestinal Research, Amsterdam Gastroenterology and Metabolism, Amsterdam, the Netherlands; 5 Department of Molecular Cancer Research, Center for Molecular Medicine, University Medical Center Utrecht, Utrecht, The Netherlands; 6 Department of Gastroenterology and Hepatology, Tianjin Medical University General Hospital, Tianjin, China; 7 Institute of Molecular Biology and Biotechnology, Bahauddin Zakariya University Multan, Multan, Pakistan

**Keywords:** Chemical genetics, Preclinical research

## Abstract

The nuclear receptor Farnesoid X Receptor (FXR) is activated by bile acids and controls multiple metabolic processes, including bile acid, lipid, carbohydrate, amino acid and energy metabolism. Vitamin A is needed for proper metabolic and immune control and requires bile acids for efficient intestinal absorption and storage in the liver. Here, we analyzed whether FXR regulates vitamin A metabolism. Compared to control animals, FXR-null mice showed strongly reduced (>90%) hepatic levels of retinol and retinyl palmitate and a significant reduction in lecithin retinol acyltransferase (LRAT), the enzyme responsible for hepatic vitamin A storage. Hepatic reintroduction of FXR in FXR-null mice induced vitamin A storage in the liver. Hepatic vitamin A levels were normal in intestine-specific FXR-null mice. Obeticholic acid (OCA, 3 weeks) treatment rapidly reduced (>60%) hepatic retinyl palmitate levels in mice, concurrent with strongly increased retinol levels (>5-fold). Similar, but milder effects were observed in cholic acid (12 weeks)-treated mice. OCA did not change hepatic LRAT protein levels, but strongly reduced all enzymes involved in hepatic retinyl ester hydrolysis, involving mostly post-transcriptional mechanisms. In conclusion, vitamin A metabolism in the mouse liver heavily depends on the FXR and FXR-targeted therapies may be prone to cause vitamin A-related pathologies.

## Introduction

The Farnesoid X Receptor (FXR/*NR1H*) is a pleiotropic ligand-activated nuclear receptor controlling a great variety of cellular processes, including energy metabolism, immunomodulation and tissue regeneration. Bile acids are the natural ligands for FXR and these molecules are increasingly recognized as key signaling molecules controlling whole body homeostasis, much more than the original view of being just detergents for lipid-soluble compounds^1^. FXR typically forms a heterodimer with the Retinoid X Receptor-alpha (RXRα/*NR2B1*)), a common obligatory partner for many nuclear receptors, including peroxisome proliferator-activated receptors (PPARs), Liver X receptor (LXR), Retinoic acid receptor (RAR) and the vitamin D receptor (VDR). RXRα is activated by retinoic acids, particularly 9-cis retinoic acid (9cRA), the active metabolites of vitamin A^2^. RXRα is not a silent partner of FXR. Co-activation of FXR/RXRα with 9cRA may have strong transcriptional effects of FXR-target genes. This can go either way: e.g. further enhancing bile acid-induced expression, as for the small heterodimer partner (SHP/*NR0B2*)^3^, or strongly suppressing it, as for the bile salt export pump (BSEP/*ABCB11*)^4,5^. Moreover, retinoic acids suppress Cyp7A1 expression, the rate limiting factor in hepatic bile acid synthesis, through direct transcriptional effects in the liver, as well as by inducing expression of intestinal fibroblast growth factor 15/19 (mouse FGF15/human FGF19), which in turn suppresses hepatic bile acid synthesis^6–8^.

Vitamin A deficiency is a common condition in chronic liver diseases. This is mainly the result of 2 pathological processes, e.g. cholestasis and fibrosis. Vitamin A is a fat-soluble vitamin and its intestinal absorption depends on bile acids^9^. Cholestasis is characterized by insufficient bile flow from the liver and thereby impairs intestinal vitamin A absorption. Absorbed vitamin A is efficiently transported to the liver, where it is stored as retinyl esters, mainly retinyl palmitate, in hepatic stellate cells^10^. The “quiescent” HSC (qHSC) in the healthy liver maintain stable circulating levels of retinol at approximately 1.5–2.0 µmol/L to be delivered to peripheral tissues to support proper function^11^. Liver injury in chronic liver diseases, however, induce a phenotypic change in HSC that transdifferentiate to contractile, mobile and extracellular matrix-producing myofibroblasts, so-called activated HSC (aHSC). The qHSC-to-aHSC transdifferentiation process is the driving force for the development of fibrosis and characterized by progressive loss of the retinyl ester stores from these cells, ultimately leading to hepatic an systemic vitamin A deficiency^9,12^.

While the vitamin A-mediated regulation of bile acid synthesis and transport is well established^3,5–9^, very little is known about a putative role of FXR in regulating hepatic vitamin A metabolism. Key factors for hepatic vitamin A metabolism are 1) the LDL receptor in hepatocytes, which absorbs retinyl ester-containing chylomicron remnants coming from the gut; 2) Retinyl hydrolases (ATGL, PNPLA3, amongst others) in hepatocytes that convert retinyl ester to retinol; 3) Retinol Binding Protein 4 (RBP4) that exports retinol from the hepatocytes to the circulation; 4) retinyl esterases (LRAT and DGAT1) in hepatic stellate cells that convert retinol to retinyl esters for storage; 5) Retinyl hydrolases (ATGL, PNPLA3, HSL) in HSC for controlled release of retinol in times of insufficient dietary intake; 6) RDHs and RALDHs that convert retinol to retinoic acids and 7) cytochrome P450s (particularly Cyp26A1) that catabolize retinoic acids (see also Saeed 2017 BBA)^9^. Previous transcriptome analysis have not hinted to a clear effect of FXR on hepatic vitamin A metabolism^13–15^. Such FXR-mediated transcriptional effects have been well-established for regulation of hepatic lipid and glucose metabolism^13–16^. The pharmacological FXR agonist obeticholic acid (OCA; 6α-ethyl-chenodeoxycholic acid; INT-747) is used for the treatment of Primary biliary cholangitis (PBC), either as mono therapy or in combination with ursodeoxycholic acid (UDCA)^17–19^. In addition, OCA and several other pharmacological FXR ligands are being evaluated in clinical trials for their therapeutic value in other chronic liver diseases, including primary sclerosing cholangitis (PSC) and non-alcoholic fatty liver disease (NAFLD). As vitamin A metabolism is typically impaired in chronic liver diseases like PBC, PSC and NAFLD^20–24^, FXR may also play a role in this, either directly or indirectly.

In this study, we therefore analyzed vitamin A metabolism in total and tissue-specific FXR-null mice, all on normal chow, as well as in WT mice treated with OCA, or cholic acid (CA) to dissect the effects of FXR in vitamin A metabolism. To our surprise, we found that both the absence of hepatic FXR, as well as the pharmacological activation by OCA, lead to vitamin A depletion from the liver. Thus, proper control of vitamin A metabolism in the liver depends on a tightly-balanced action of FXR.

## Results

### Hepatic fat accumulation, but vitamin A depletion in FXR null mice

**Figure 1:**
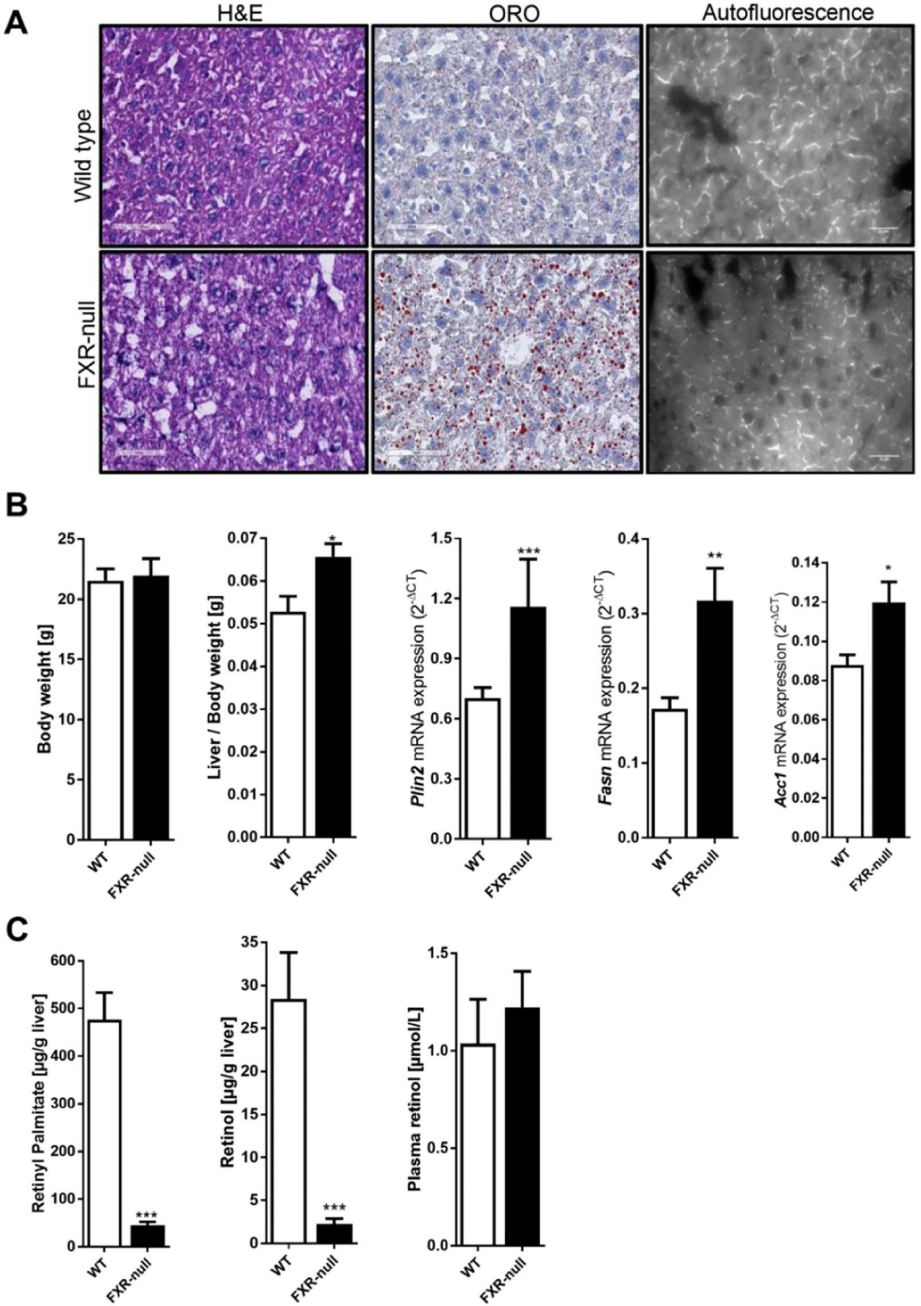
FXR deficiency impairs hepatic retinoid storage. Whole body FXR-null mice and age-match wild-type control mice were analyzed for (**A**) H&E and oil red O staining, which revealed fat accumulation in livers of FXR-null mice as compared to control mice, while autofluorescence analysis revealed reduced vitamin A levels in livers of FXR-null mouse. (**B**) The body weight was not altered FXR-null mice, while the liver weight was increased in FXR-null mice. As expected, hepatic expression of *Plin2*, *Fasn* and *Acc1* were increased in FXR-null mice vs wild-type mice. (**C**) hepatic retinyl palmitate and retinol levels were reduced in FXR-null mice, while plasma retinol levels were equal in both groups. Data are presented as Mean ± SEM and mRNA expression of genes is presented in 2^−delta CT^, which was normalized to *36B4*.

FXR-null mice have previously been shown to develop mild steatohepatitis, particularly accumulating triglycerides, which may progress to NASH even when fed a chow diet^25–29^. Indeed, Oil-Red-O staining of liver tissue of 10–12 week-old FXR-null mice demonstrated fat accumulation compared to their wild type litter mates (Fig. 1A, middle panels). Moreover, liver weight was slightly increased in FXR-null mice and showed enhanced mRNA levels of genes involved in lipogenesis, such as *Plin2*, *Fasn* and *Acc1* **(**Fig. 1B**)**. In sharp contrast and unexpectedly, hepatic retinyl palmitate and retinol levels were strongly (>90%) reduced in FXR-null mice compared to their WT littermates (Fig. 1C). Lower hepatic vitamin A levels in FXR-null mice were also confirmed by reduced vitamin A-specific autofluorescence in liver tissue (Fig. 1A, right panels). The sharp depletion of hepatic vitamin A was, however, not accompanied by a reduction in plasma retinol levels in FXR-null mice (Fig. 1C). This is not necessarily surprising as mice on a vitamin A-deficient diet are also able to maintain normal serum retinol levels, even if the liver contains less than 5% retinyl esters compared to animals on a vitamin A-containing diet (Supplementary Figure S1). Still, these results show that hepatic retinoid levels are drastically decreased in mice lacking FXR.

### Hepatic and not intestinal FXR is essential for hepatic vitamin A storage

**Figure 2:**
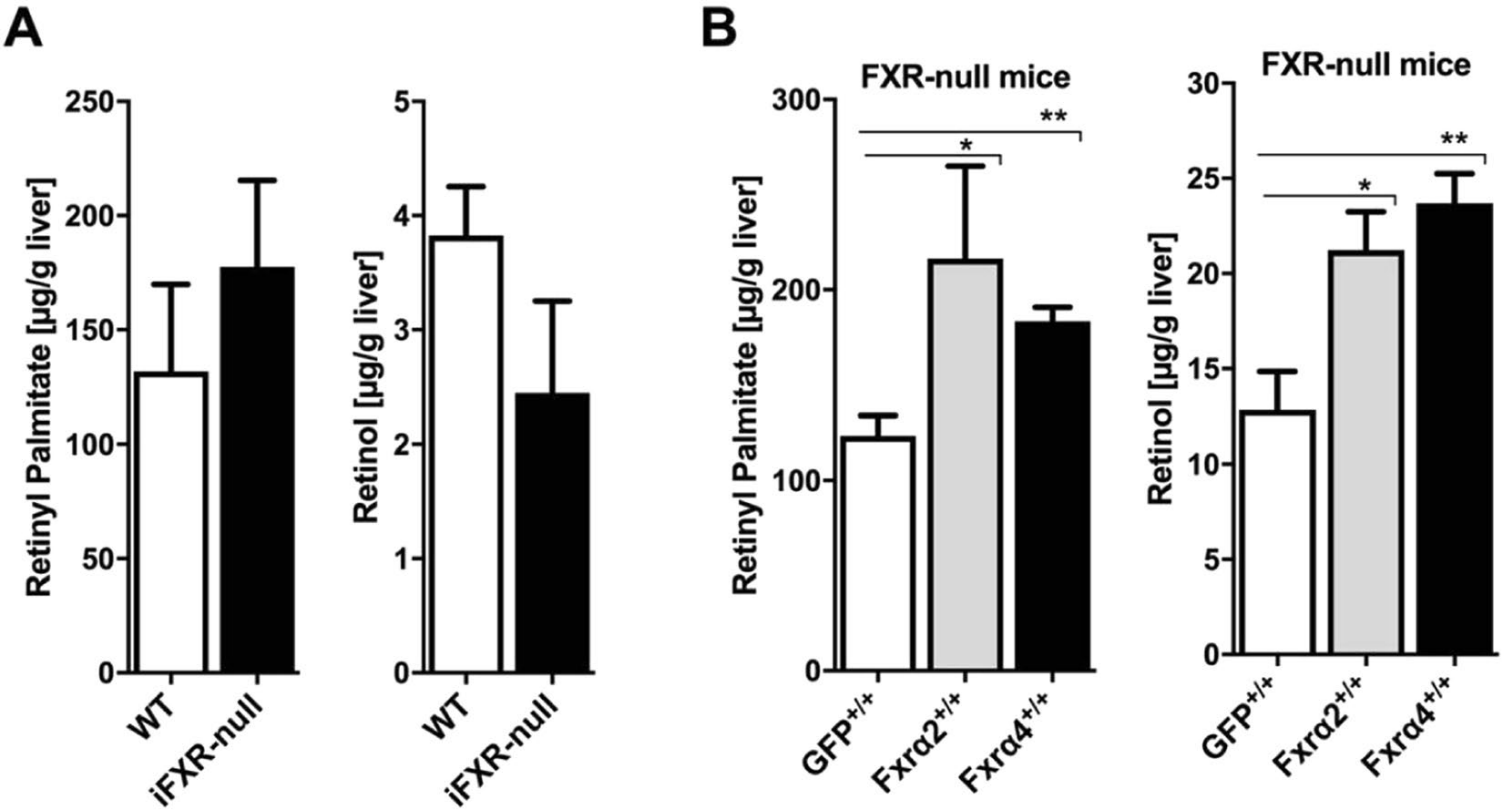
Hepatic and not intestinal FXR is essential for normal retinoid levels in the liver. (**A**) Hepatic retinyl palmitate and retinol were analyzed in intestinal specific FXR-null mice (iFXR-null) and wild-type littermates. Intestine-specific FXR-deficiency did not alter hepatic vitamin A levels. (**B**) Whole body FXR-null mice were transduced with ScAAV-produced FXRα2 or FXRα4 or GFP controls and after 4 weeks analyzed for hepatic retinyl palmitate and retinol levels. Both FXR-isoform enhanced hepatic retinoid levels, suggesting that hepatic FXR plays a crucial role in vitamin A storage and metabolism.

As dietary vitamin A is absorbed in the intestine and transported to the liver for storage, we next studied whether hepatic and/or intestinal FXR is controlling hepatic vitamin A levels. Hepatic retinyl palmitate and retinol levels were not significantly different in intestine-specific FXR-null mice (iFXR-null) as compared to WT control animals **(**Fig. 2A), suggesting that vitamin A metabolism is normal in these mice. In contrast, reintroduction of hepatic FXRα2 or FXRα4 in FXR-null mice through adenoviral expression (4 weeks) elevated both retinyl palmitate and retinol levels in mouse livers liver **(**Fig. 2B**)**. As FXR expression was driven by the hepatocyte-specific *Lp1* promoter^30,31^, these data also suggest that particularly hepatocytes play a key role in the FXR-mediated control of hepatic vitamin A levels.

### Post-transcriptional reduction of retinol esterifying LRAT in FXR-null mice

**Figure 3:**
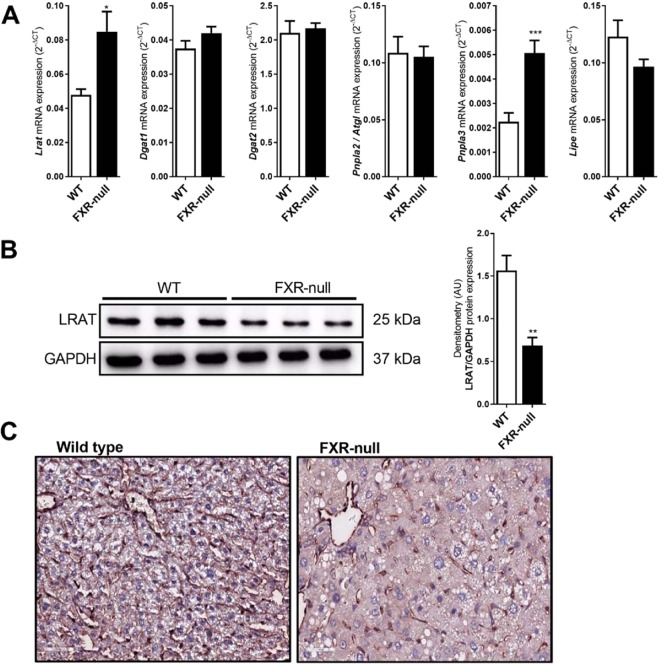
Post-transcriptional reduction of LRAT in FXR-null mice. Whole body FXR-null and wild type mice were analyzed for (**A**) hepatic mRNA expression of *Lrat, Dgat1, Dgat2, Pnpla2 (Atgl), Pnpla3, Lipe (Hsl*). Hepatic *Lrat* and *Pnpla3* mRNA levels were increased in FXR-null mice, with no change in other vitamin A metabolizing enzymes as compared to control. Western blot analysis (**B**) and immunohistochemical staining (**C**) for LRAT revealed that hepatic protein levels of LRAT were significantly reduced in FXR-null mice as compared to wild-type control mice. Data are presented as Mean ± SEM and mRNA expression of genes is presented in 2^−delta CT^, which was normalized to *36B4*.

Hepatic retinyl ester and retinol levels are a resultant of the activity of retinyl esterases (LRAT and to a lesser extent by DGAT1 and 2) and retinyl ester hydrolases (ATGL, PNPLA3 and HSL). mRNA levels of *Dgat1* and 2, *Pnpla2* (ATGL) and *Lipe* (HSL) were comparable between FXR-null mice and WT littermates, while both *Lrat* and *Pnpla3* levels were elevated in FXR-null mice (Fig. 3A). As the latter may hint to a more dynamic vitamin A metabolism in the liver, it does not provide a clear explanation for the depletion of hepatic retinyl esters and retinol in FXR-null mice. Detailed (re)analysis of public transcriptome analysis comparing wild-type and FXR-null mice also did not reveal a prominent effect on genes involved in hepatic vitamin A metabolism when FXR is absent **(**Supplementary Figure S2**)**. As LRAT plays a key role in retinyl ester accumulation in the liver, we analyzed the protein level in more detail. In contrast to the elevated *Lrat* mRNA levels, hepatic LRAT protein levels were significantly reduced (>60%) in FXR-null mice compared to WT littermates, as quantified by Western blotting (Fig. 3B). In line, LRAT-specific immunohistochemical staining was strongly reduced in liver sections of FXR-null mice compared to WT animals, while a stellate cell-specific staining was preserved (Fig. 3C). These data reveal a post-transcriptional effect caused by the absence of FXR that may aid to the impaired accumulation of hepatic retinoids.

### OCA-mediated activation of FXR reduces hepatic vitamin A levels

**Figure 4:**
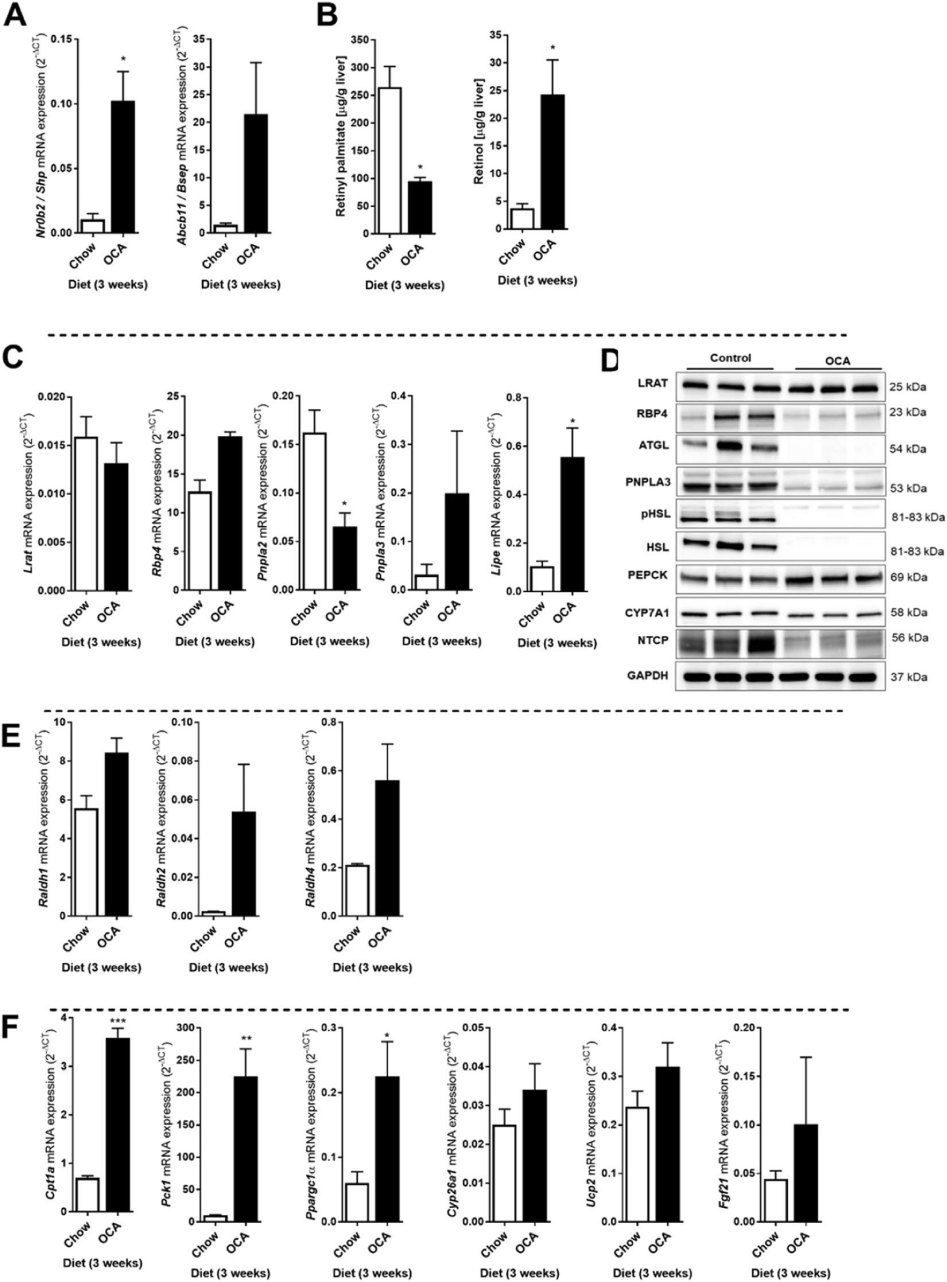
OCA treatment reduces hepatic retinyl palmitate, while increasing retinol levels in mouse livers. Control and OCA-treated mice were analyzed for hepatic vitamin A levels, mRNA and protein levels of FXR targets and vitamin A metabolizing factors. (**A**) The mRNA levels of FXR target genes *Nr0b2* (encoding SHP) *and Abcb11* (encoding BSEP) were strongly enhanced in livers of OCA-treated mice. (**B**) Hepatic retinyl palmitate levels were significantly reduced in OCA-treated mice, while hepatic retinol levels were significantly increased as compared to control mice. (**C**) Hepatic mRNA levels of *Lrat, Rbp4 and Pnpla3* were not changed, while levels of *Pnpla2* (ATGL) were decreased and *Lipe* (HSL) were increased in OCA-treated mice. (**D**) Protein levels of LRAT were not affected by OCA-treatment, while RBP4, ATGL, PNPLA3, HSL (and its active phosphorylated form pHSL), CYP7A1 and NTCP were all decreased and PEPCK was increased. GAPDH was used as loading control. (**E**) mRNA levels of genes involved in the conversion of retinol into retinoic acid (*Raldh1, Raldh2, Raldh4*) and of (**F**) retinoic acid-responsive genes (*Cpt1a, Pck1, Ppargc1a, Cyp26a1, Ucp2, Fgf21*) were enhanced in OCA-treated mice as compared to control animals. Data are presented as Mean ± SEM and mRNA expression of genes is presented in 2^−delta CT^, which was normalized to *36B4*.

Given the pronounced reduction of hepatic retinoids in the absence of FXR in mouse livers, we next analyzed the effect of pharmacological activation of FXR in WT mice. Mice were treated for 3 weeks with obeticholic acid (OCA). As expected, mRNA levels of the FXR target genes *Nr0b2* (Shp) and *Abcb11* (Bsep) were enhanced by OCA treatment (Fig. 4A). In contrast to our expectation, OCA treatment also strongly decreased hepatic retinyl palmitate levels (−64%) compared to control animals, while retinol concentrations were sharply elevated (+5-fold) in the liver **(**Fig. 4B). mRNA levels of *Lrat* and *Rbp4* were not affected by OCA treatment, while a pronounced induction was observed for the genes encoding the retinyl ester hydrolases PNPLA3 and HSL (*Lipe*), concomitant with a reduction in *Pnpla2* (ATGL) mRNA levels **(**Fig. 4C). Surprisingly, however, Western blot analysis revealed that protein levels of all 3 hepatic retinyl ester hydrolases (ATGL, PNPLA3 and HSL, including its active, phosphorylated form pHSL) were drastically decreased in OCA-treated animals (Fig. 4D), while LRAT levels were not changed compared to control animals. The high retinol levels in the livers of OCA-treated animals (Fig. 4B) are therefore unlikely to come from hydrolysis of hepatic retinyl esters. Interestingly, hepatic RBP4 protein levels appeared lower in OCA-treated animals, while *Rbp4* mRNA levels tended to be increased, which may be a result of retinol-induced release from the liver^32–34^. Importantly, OCA did lead to expected induction of PEPCK (encoded by *Pck1*) and reductions in CYP7A1 and NTCP (Fig. 4D). To examine whether hepatic retinol metabolism to retinoic acids was blocked, we analyzed expression of RALDHs and found that mRNA levels of *Raldh1*, 2 and 4 were induced by OCA **(**Fig. 4E**)** and was accompanied by increased levels of retinoic acid-responsive genes, like *Cpt1a, Pck1, Ppargc1a, Cyp26a1, Ucp2, Fgf21* **(**Fig. 4F). Taken together, these data show that, even though the capacity to esterify retinol seems to be increased by OCA (LRAT maintained, while retinyl hydrolases are strongly reduced), it enhances hepatic retinol levels while retinyl ester stores go down. Moreover, the production of retinoic acids in the liver seems not impaired or even enhanced in OCA-treated animals.

### Cholic acid feeding also impairs retinyl ester accumulation in mouse liver

**Figure 5:**
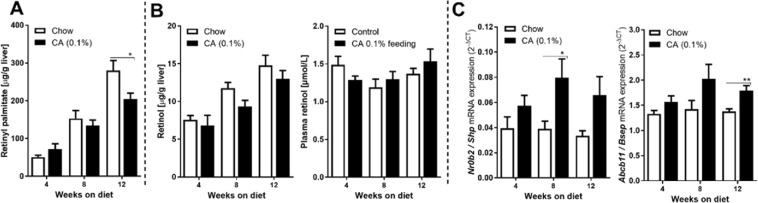
Cholic acid (0.1%) treatment delays the accumulation of hepatic retinoids in mice. Mice were fed chow or a CA (0.1%)-containing diet for 4, 8 and 12 weeks. (**A**) Hepatic retinyl palmitate was significantly decreased CA-fed treated mice after 12 weeks as compared to control mice. (**B**) Hepatic and serum retinol levels did not differ at any time point between both groups of mice. (**C**) Hepatic mRNA levels FXR-responsive genes *Nr0b2* (SHP) and *Abcb11* (BSEP) were moderately increased in CA-treated animals compared to control mice. Data are presented as Mean ± SEM and mRNA expression of genes is presented in 2^−delta CT^, which was normalized to *36B4*.

Given the remarkable observation that both the absence and pharmacological activation of FXR leads to reduction of retinyl ester levels in the liver, we also analyzed whether cholic acid treatment may affect hepatic vitamin A metabolism in mice. Mice were fed a diet containing 0.1% CA for up to 12 weeks. Both retinyl palmitate and retinol levels in the liver increase with age when animals are fed normal (vitamin A-containing) chow, as has been reported before^35,36^ No significant differences in retinyl palmitate levels were detected after 4 or 8 week CA feeding compared to animals on control chow, although the accumulation of hepatic retinyl palmitate seemed delayed between week 4 and 8 in CA-fed mice. This indeed progressed to a moderate (−29%), but significant reduction in retinyl palmitate after 12 weeks feeding a CA-containing diet, compared to control animals (Fig. 5A). At none of the time points an effect of the CA diet on hepatic and serum retinol levels was observed (Fig. 5B). The CA-diet induced the hepatic expression of of *Nr0b2* (Shp) and *Abcb11* (Bsep) (Fig. 5C), though the effect was less pronounced compared to OCA (Fig. 4A).

Thus, all together, our data show that both the absence and activation of FXR impairs hepatic vitamin A storage.

## Discussion

In this study, we report the remarkable observation that both the absence, as well as ligand-activation of FXR leads to a reduction in hepatic retinyl ester stores in mice. FXR presence in the liver appears essential for promoting retinyl ester accumulation, while intestinal FXR is not. Pharmacological activation of FXR by OCA left hepatic LRAT expression unaffected, while it strongly suppressed levels of hepatic retinyl ester hydrolases (ATGL, PNPLA3 and HSL) by combined transcriptional and non-transcriptional mechanisms. The accompanied retinyl ester depletion and retinol accumulation appears therefore not (solely) a result of impaired vitamin A metabolism inside the liver. FXR is an important therapeutic target for liver disease and is currently evaluated in clinical trials for the treatment of chronic liver diseases, including PSC and NASH^37,38^. Effects of FXR ligands on vitamin A metabolism therefore deserve attention, as vitamin A metabolites serve many key physiological functions disturbed in liver disease, such as in immune regulation, tissue differentiation, bile acid, glucose and fat metabolism.

To the best of our knowledge, this is the first study that analyzed the putative role of FXR in vitamin A metabolism. FXR has already been shown to control diverse metabolic processes, varying from bile acid, triglyceride, cholesterol, glucose to amino acid metabolism^39–42^. In addition, it regulates autophagy, inflammation and extracellular matrix production^43–45^. A direct effect of FXR on transcriptional regulation of genes involved in these processes has been confirmed^39–46^. In this study, we have so far been unable to make such direct link with FXR-controlled gene expression for vitamin A metabolism. We have re-evaluated public databases with micro-array and RNAseq data on FXR-mediated effects, but have not discovered clear overlooked genes involved in vitamin A metabolism. Both the absence of FXR and OCA-treatment in this study lead to expected transcriptional effects on hepatic genes involved in lipid (Fig. 1B) and bile acid (Fig. 4A) metabolism, respectively. With respect to the genes involved in vitamin A metabolism, FXR absence enhanced mRNA levels of *Lrat* while reducing *Pnpla3* and leaving *Pnpla2* and *Lipe* levels unchanged. While this may suggest an enhanced metabolism back and forth between retinyl esters and retinol, it does not push a direction to retinyl ester and/or retinol loss from the liver. In contrast to the enhanced *Lrat* mRNA levels, LRAT protein levels were actually decreased in FXR-null mice. LRAT-null mice are unable to store retinyl esters in the liver^47^, thus a significant reduction in LRAT protein may impair retinyl ester storage and aid to an explanation for the reduced retinyl ester levels in FXR-null mice. However, this appears not a result of FXR-mediated transcriptional regulation as *Lrat* mRNA levels were unaffected by the absence of FXR in the liver. Analysis of hepatic mRNA levels after 3 week OCA treatment provided some hints for the rapid change in retinyl ester (>60% down) and retinol (>5-fold up) levels in the liver, where enhanced mRNA levels of *Pnpla3* and *Lipe* were detected with stable *Lrat* mRNA levels. Surprisingly, however, corresponding protein levels of enzymes known to hydrolyse retinyl esters (ATGL, PNPLA3 and HSL) were all strongly reduced in the liver. Thus, as observed in FXR-null mice, post-transcriptional effects associated with OCA treatment appear to have pronounced effects on vitamin A metabolism. At present, we cannot explain the high hepatic retinol levels induced by OCA with the transcriptional and post-translational regulatory mechanisms in the liver. OCA treatment may affect the bile acid composition in the intestine and, as a consequence, efficient intestinal vitamin A absorption. Still, the OCA-induced changes in the biliary, intestinal and fecal bile acid pool are reported to be relatively mild^48,49^, which would not majorly impair intestinal vitamin A absorption. Importantly, blockade of intestinal vitamin A absorption lowers both retinyl ester and retinol levels in the liver, as observed in mice on a VAD diet (Supplementary Figure S1). Thus, the remarkable increase in hepatic retinol levels after OCA treatment is not likely to be primarily a result of vitamin A malabsorption. One hypothesis to be tested is whether the high retinol levels in the liver may actually arise from impaired vitamin A metabolism in the intestinal epithelium. This requires a detailed analysis of vitamin A metabolites in the intestine and circulation, samples that were unfortunately not available for this study.

The bile acid pool and composition are also altered in whole body- and tissue-specific FXR-null mice compared to control mice^49–51^. However, as for OCA-treated mice, such changes appear not so severe that it will almost fully impair absorption of fat-soluble vitamin A^48,49^. In fact, the total bile acid pool is increased in whole body- and liver-specific FXR-null mice, and fecal bile acid concentrations were not reduced^50,51^, making it unlikely that an effect on intestinal absorption is the driving force for the pronounced depletion of hepatic vitamin A levels in FXR-null mice. Unfortunately, intestinal vitamin A absorption is experimentally difficult to assess quantitatively in *in vivo*/*ex vivo* experiments. Retinyl esters are first converted to retinol in the intestinal lumen, transported across the apical membrane of enterocytes, reconverted back to retinyl esters and incorporated in chylomicrons and secreted in the circulation. Apart from this, also some retinol may be secreted directly by enterocytes to the circulation^9^. Pulse-chase experiments with labeled retinoids would be needed to compare intestinal vitamin A absorption efficiency in WT versus FXR-null animals and/or the effect of OCA treatment^52,53^. Our results make such experiments relevant to perform.

In contrast to a potential effect of OCA on intestinal vitamin A metabolism leading to a strongly disturbed retinyl ester/retinol balance in the liver, hepatic FXR seems to be critically important to maintain normal levels of hepatic retinyl esters and retinol. Previously, adenoviral reintroduction of the FXR isoforms α2 and α4 in FXR-null mice was shown to enhance mRNA levels of various lipases (incl. *Pnpla2* and *Lipe*, encoding ATGL and HSL respectively) and decrease hepatic triglyceride levels^54^. Interestingly, ATGL and HSL hydrolyse both triglycerides and retinyl esters. Still, hepatic retinyl ester stores increase with reintroduction of hepatic FXR (this study) while triglycerides decrease^54^. On the other hand, hepatic retinol levels go up after reintroduction of Fxra2 and a4, which could be a result of the enhanced *Pnpla2* and *Lipe* levels. Given the fact that protein levels of these lipases may strongly deviate from the corresponding mRNA levels, as we observed in the OCA-treated animals, it remains to be determined what is the driving force of the triglyceride decline and vitamin A increase in these animals.

Some of the hepatic effects observed earlier in the FXR-null mice after reintroduction of FXR may actually be caused directly or indirectly by the changed hepatic vitamin A metabolism. Retinoic acids regulate hepatic lipid metabolism at various levels by activating RARs and RXRs, either directly or in complex with other nuclear receptors, like PPARa, PPARg, LXR and also FXR. All-trans retinoic acids, typically activating RARs, have been shown to suppress hepatic steatosis^55^. Activation of RXR by 9-cis retinoic acid or pharmacological ligands has a less-predictable outcome, as it co-activates nuclear receptors promoting lipolysis (PPARa, FXR)^56–58^, as well as those that promote lipogenesis (PPARg, LXR)^59,60^. It will therefore greatly depend on local levels of the various nuclear receptors and the type of retinoic acid produced which process will dominate, either lipolysis or lipogenesis. Thus, the therapeutic action of FXR ligands, including OCA, may in part be due to effects on hepatic vitamin A metabolism.

Not only OCA, but also CA-feeding impaired hepatic vitamin A storage in mice, which was significantly reduced after 12 weeks on a diet containing 0.1% CA. This concentration was chosen as it resembles the mouse-equivalent of the therapeutic dose as FDA-approved bile acids for the treatment of several liver diseases, including gallstones, inborn errors of bile acid synthesis and peroxisomal disorders (Zellweger syndrome)^61–63^. Particularly, the age-dependent increase in hepatic retinyl palmitate levels was suppressed in mice on a CA-containing diet although the absolute effect was much less pronounced as compared to the 3 week OCA treatment. Still, it may be recommended to regularly check patients on bile acid therapy for circulating retinol levels. Although serum retinol levels do not accurately represent the hepatic pool of vitamin A, they will drop at the stage when the liver gets depleted from vitamin A. According to our results, hepatic vitamin A depletion is likely to develop more rapidly under OCA treatment. It is important to note that the OCA therapy is already available for PBC and in clinical trials for other chronic liver diseases that are known to be associated with vitamin A deficiency, including PBC, PSC and NASH. OCA treatment may therefore accelerate vitamin A depletion in these patients affecting proper immune regulation and metabolic control. Future studies need to address whether patients treated with therapeutic bile acids or FXR-ligands are truly at risk for conditions caused by impaired vitamin A metabolism.

An important limitation of our study is the fact that the various animal experiments were performed at 4 different laboratories with different suppliers of the chow. As hepatic retinoid levels are strongly dependent on the composition of the diet and the age of the animals, levels of hepatic retinyl palmitate and retinol varied significantly between the various control groups. The amount of vitamin A in the chow was 10 IU of vitamin A per gram chow in the genetic models and OCA-treatment experiment, while the CA-treatment experiment contained 4 IU vitamin A/g. It was therefore crucial that each experiment had the proper animal control group included, where the hepatic retinyl palmitate and retinol levels differed significantly between these control groups. Importantly, all vitamin A measurements were performed in the same laboratory by the same researchers to exclude analytical differences. On the other hand, the fact that a clear effect of FXR and its ligands on hepatic retinoid levels was observed in all animal experiments also underscores the fact that a clear relationship seems to exist between FXR and vitamin A metabolism, although this may not be regulated primarily at the transcriptional level. This, in fact, uncovers a completely unexplored research field of the possible post-transcriptional effects associated with manipulation of FXR activity.

In conclusion, our data show that the absence of hepatic FXR, as well as the oral supplementation of natural and synthetic bile acids impair vitamin A metabolism in the liver. Vitamin A serves a great variety of physiological functions in and outside the liver, including immune regulation and metabolic control. Future studies therefore need to address whether the therapeutics targeting FXR may lead to vitamin A-related adverse effects.

## Materials and Methods

### Animal experiments

Animal experiments were performed after the approval by institutional animal care and use committee of Universities of Groningen, institutional animal care and use committee of University of Amsterdam and institutional animal care and use committee of University Medical Center Utrecht, The Netherlands and institutional animal care and use committees of Tianjin Medical University, China. All animal experiments were performed in accordance with the relevant guidelines and regulations. All animals were kept in a pathogen-free control environment with alternating dark and light cycles of 12 hours, temperature (20–24 °C) and relative humidity (55% ± 15%). Animals received food and water *ad libitum*.

FXR-null mice mice^25,64^ (data presented in Figs. 1 & 3) or intestinal specific FXR-null mice (iFXR-null) mice^65^ (data presented in Fig. 2A) and wild-type mice were kept on standard chow diet (RMHB; Hope Farms, The Netherlands; contains 10 IU of retinyl acetate/g as source of vitamin A) for 8–10 weeks, and then sacrificed for analysis. FXRα2 and FXRα4 isoforms were reintroduced in livers of FXR-null mice as described previously^66^ using self-complementary adeno-associated virus (scAAV) serotype 8 vectors under control of the liver-specific LP1-promoter. ScAAV expressing the Green Fluorescent Protein (GFP) were used as control. FXR-null mice received 1 × 10^11^ AAV particles into the retro-orbital sinus with vector genomes (data presented in Fig. 2B). All animals were sacrificed after one month of scAAV transfection for analysis, as described previously^66^. Wild type C57BL/6 mice were fed a chow diet diet (K4068.02; Arie Blok diervoeders, Woerden, The Netherlands; contains 10 IU of retinyl acetate/g as source of vitamin A) mixed with either vehicle or obeticholic acid (OCA; 0.025% w/w) for 3 weeks ad libitum^67^ (data presented in Fig. 4). Alternatively, wild type C57BL/6 mice were fed a chow diet (H10293G, Hua Fukang Biological, technology, Beijing, China; contains 4 IU of retinyl acetate/g as source of vitamin A) mixed with either vehicle or cholic acid (0.1% w/w) (Sigma-Aldrich, China, Inc) for 4, 8 or 12 weeks (data presented in Fig. 5). For all animal experiments mice were sacrificed at the end of the experiment, and liver tissue harvested for further analysis. Tissue was snap-frozen in liquid nitrogen and stored at −80 °C until further use. Additionally, tissue was fixed with 4% paraformaldehyde for (immuno)histochemical analysis. All analyses were conducted in Department of Gastroenterology and Hepatology, University Medical Center Groningen, University of Groningen, The Netherlands. Snap-frozen samples were transported according to international and European regulation of import and export and on dry ice to preserved stability of retinoids.

***Standard protocols*** for vitamin A analysis, mRNA (qRT-PCR), protein (Western blotting) and microscopy are presented in supplementary Material and Methods.

### Statistical analysis

Data are presented as Mean ± SEM and statistical analysis was performed with GraphPad Prism 6 software package (GraphPad Software, San Diego, CA, USA). Statistical significance between two groups was determined by Mann-Whitney test. P-values ≤ 0.05*,  ≤ 0.01**,  ≤ 0.001*** were considered significant.

## Supplementary information

Supplementary Information
