# Supplementary material for: Farnesoid X receptor and bile acids regulate vitamin A storage
Source: Sci Rep. 2019 Dec 20;9:19493. doi: 10.1038/s41598-019-55988-w (PMC6925179; doi:10.1038/s41598-019-55988-w)
Supplement: Supplementary file 1 — Supplementary Information [file 41598_2019_55988_MOESM1_ESM.pdf]

Farnesoid X receptor and bile acids regulate vitamin A storage

Ali Saeed[1,7$], Jing Yang[1,6], Janette Heegsma[1,2], Albert K. Groen[3], Saskia W.C. van Mil[5], Coen C. Paulusma[4], Lu Zhou[6], Bangmao Wang[6], Klaas Nico Faber[1, 2$]

[1]Department of Gastroenterology and Hepatology, [2]Laboratory Medicine, [3]Department of Pediatrics, Center for Liver, Digestive, and Metabolic Diseases, University Medical Center Groningen, University of Groningen, Groningen, The Netherlands. [4]Amsterdam UMC, University of Amsterdam, Tytgat Institute for Liver and Intestinal Research, Amsterdam Gastroenterology and Metabolism, Amsterdam, the Netherlands. [5]Department of Molecular Cancer Research, Center for Molecular Medicine, University Medical Center Utrecht, Utrecht, The Netherlands. [6]Tianjin Medical University General Hospital, Gastroenterology and Hepatology Department, China. [7]Institute of Molecular Biology and Biotechnology, Bahauddin Zakariya University Multan, Pakistan.

Running title: FXR regulates vitamin A storage

Corresponding author: Ali Saeed and Klaas Nico Faber, Dept. Hepatology & Gastroenterology, University Medical Center Groningen; Mailing address: Hanzeplein 1, 9713 GZ Groningen, The Netherlands; Tel.: +31(0)503619951, +31(0)503612364; Fax: +31(0)503619306; email: a.saeed@umcg.nl, k.n.faber@umcg.nl

Supplementary Figure S1.

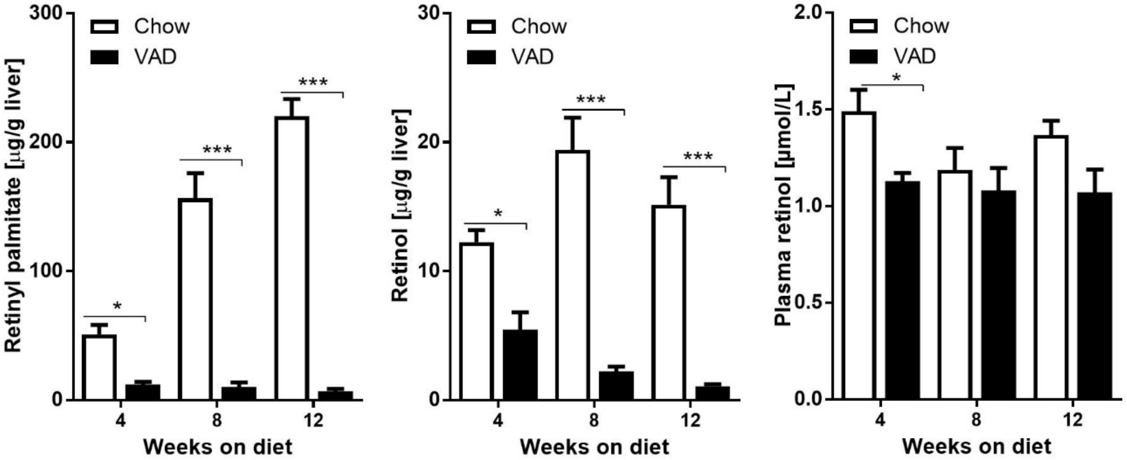

Supplementary Figure S1: **Hepatic and serum vitamin A level in mice fed-vitamin A deficient diet.** Four (4)-week old mice were fed either a chow diet (H10293G, Hua Fukang Biological, technology, Beijing, China) with 4 IU of retinyl acetate/g as a vitamin A source or a vitamin A deficient (VAD) diet (H10293G, Hua Fukang Biological, technology, Beijing, China). Hepatic Retinol and retinyl palmitate levels were analyzed after 4, 8 and 12 weeks diet. Hepatic retinyl palmitate and retinol levels were significantly reduced already after 4 weeks in mice fed a VAD diet while serum retinol levels were stable in both groups of mice during the whole course of the dietary intervention. Data are presented as Mean±SEM and mRNA expression of genes is presented in 2-delta CT which was normalized to *36B4*.

Supplementary Figure S1.

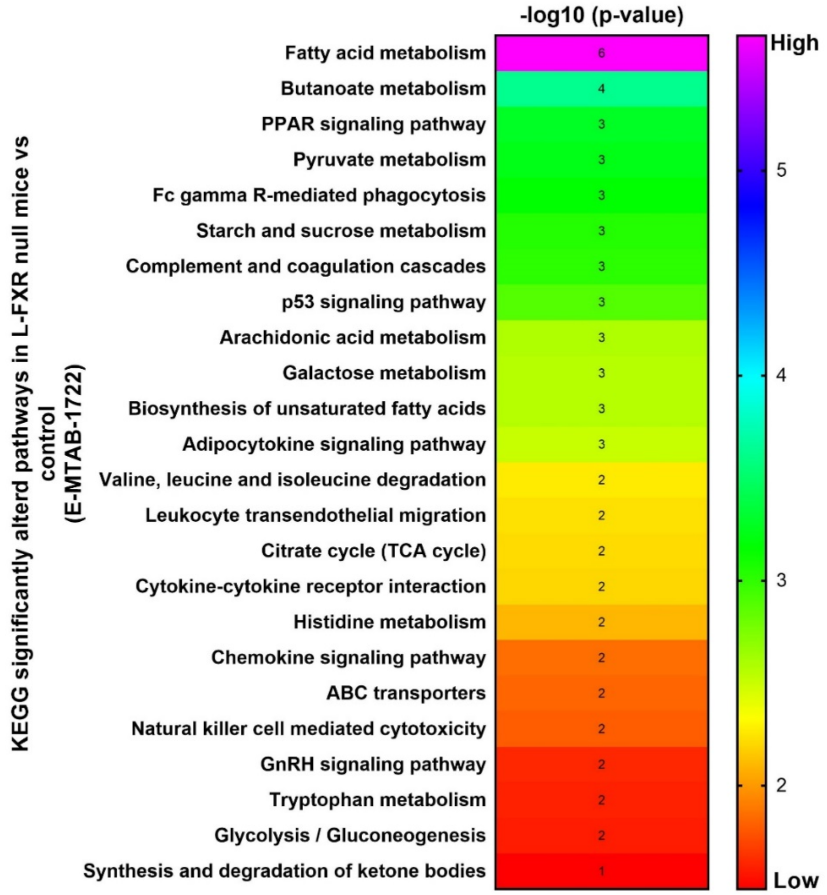

Supplementary Figure S2: **A heat map of significantly altered KEGG pathway associated with liver-specific FXR deletion.**

KEGG analysis of liver-specific FXR-null mice vs wild type mice was performed using online available DAVID 6.7 software on differentially regulated genes in publicly available microarray data (E-MTAB-1722). A heat map was generated from the –log10 p-value of the significantly altered KEGG pathways associated with liver-specific FXR-deletion vs control. Fatty acid metabolism was the top hit in this analysis, however, retinol metabolism was not significantly associated with L-FXR deletion in the liver of mice.

Supplementary Table S1: Primers and probes used in study for analysis of target genes

Gene / ID	Taqman primers and probe
36B4<br>NM_022402	Fwd: 5'-GCTTCATTGTGGGAGCAGACA-3'<br>Rev: 5'-CATGGTGTTCTTGCCCATCAG-3'<br>Probe: 5'-TCCAAGCAGATGCAGCAGATCCGC-3'
Abcb11 / Bsep<br>NM_021022.3	Fwd: 5'-CTGCCAAGGATGCTAATGCA -3'<br>Rev: 5'-CGATGGCTACCCTTTGCTTCT-3'<br>Probe: 5'-TGCCACAGCAATTTGACACCCTAGTTGG-3'
Acaca / Acc1<br>NM_133360.1 / NM_022193.1	Fwd: 5'-GCCATTGGTATTGGGGCTTAC-3'<br>Rev: 5'-CCCGACCAAGGACTTTGTTG-3'<br>Probe: 5'-CTCAACCTGGATGGTTCTTTGTCCCAGC-3'
Cpt1a<br>NM_013495.1	Fwd: 5'-CTCAGTGGGAGCGACTCTTCA-3'<br>Rev: 5'-GGCCTCTGTGGTACACGACAA-3'<br>Probe: 5'-CCTGGGGAGGAGACAGACACCATCCAAC-3'
Cyp26a1<br>NM_007811.1	Fwd: 5'-GGAGACCCTGCGATTGAATC-3'<br>Rev: 5'-GATCTGGTATCCATTCAGCTCAAA-3'<br>Probe: 5'-TCTTCAGAGCAACCCGAAACCCTCC-3'
Dgat1<br>NM_010046.2 / NM_053437.1	Fwd: 5'-GGTGCCCTGACAGAGCAGAT-3'<br>Rev: 5'-CAGTAAGGCCACAGCTGCTG-3'<br>Probe: 5'-CTGCTGCTACATGTGGTTAACCTGGCCA-3'
Dgat2<br>NM_026384.2 /<br>NM_001012345.1	Fwd: 5'-GGGTCCAGAAGAAGTTCCAGAAG-3'<br>Rev: 5'-CCCAGGTGTCAGAGGAGAAGAG-3'<br>Probe: 5'-CCCCTGCATCTTCCATGGCCG-3'
Fasn<br>NM_007988 / NM_017332	Fwd: 5'-GGCATCATTGGGCACTCCTT-3'<br>Rev: 5'-GCTGCAAGCACAGCCTCTCT-3'<br>Probe: 5'-CCATCTGCATAGCCACAGGCAACCTC-3'
Fgf21<br>NM_020013.4 / NM_130752.1	Fwd: 5'-CCGCAGTCCAGAAAGTCTCC-3'<br>Rev: 5'-TGACACCCAGGATTTGAATGAC-3'<br>Probe: 5'-CCTGGCTTCAAGGCTTTGAGCTCC A-3'
Lipe / HSL<br>NM_010719 / X51415	Fwd: 5'-GAGGCCTTTGAGATGCCACT-3'<br>Rev: 5'-AGATGAGCCTGGCTAGCACAG-3'<br>Probe: 5'-CCATCTCACCTCCCTTGGCACACAC-3'
Lrat<br>NM_023624	Fwd: 5'-TCCATACAGCCTACTGTGGAACA-3'<br>Rev: 5'-CTTCACGGTGTCATAGAACTTCTCA-3'<br>Probe: 5'-ACTGCAGATATGGCTCTCGGATCAGTCC-3'
Nr0b2 / Shp	Fwd: 5'-CCTTCTGGAGCCTGGAGCTTA -3'<br>Rev: 5'-CTGGCACATCGGGGTTGA-3'<br>Probe: 5'-ATGGTCCCTTTCAGGCAGGCATATTCCTT-3'
Pck1<br>NM_011044 / NM_198780	Fwd: 5'-GTGTCATCCGCAAGCTGAAG-3'<br>Rev: 5'-CTTTCGATCCTGGCCACATC-3'<br>Probe: 5'-CAACTGTTGGCTGGCTCTCACTGACCC-3'
Plin2<br>NM_175640.2 /<br>NM_001113471.1	Fwd: 5'-AGAACGTGCTCAGAGAGGTTACAG-3'<br>Rev: 5'-GTGTTCTGCACGGTGTGTACC-3'<br>Probe: 5-CCTGCCCAACCCGAGAGGCC-3'
Ppargc1 α / Pgc1α<br>NM_008904 / NM_031347	Fwd: 5'-GACCCCAGAGTCACCAAATGA-3'<br>Rev: 5'-GGCCTGCAGTTCCAGAGAGT-3'<br>Probe: 5'-CCCCATTTGAGAACAAGACTATTGAGCGAACC-3'
Pnpla2 / Atgl<br>NM_025802 / XM_347183	Fwd: 5'-AGCATCTGCCAGTATCTGGTGAT-3'<br>Rev: 5'-CACCTGCTCAGACAGTCTGGAA-3'<br>Probe: 5'-ATGGTCACCCAATTTCCTCTTGGCCC-3'
Pnpla3<br>NM_054088	Fwd: 5'-ATCATGCTGCCCTGCAGTCT-3'<br>Rev: 5'-GCCACTGGATATCATCCTGGAT-3'<br>Probe: 5'-CACCAGCCTGTGGACTGCAGCG-3'
Raldh1	Assay on demand, Mm00657317_m1 (ThermoFisher)
Raldh2	Assay on demand, Mm00501306_m1 (ThermoFisher)
Raldh4<br>NM_178713.4	Fwd: 5'-TGGAGCAGTCTCTGGAGGAGTT-3'<br>Rev: 5'- GAAGTTCAGAACAGACCGAGGAA-3'<br>Probe: 5'- AATCTAAAGACCAAGGGAAAACCCTCACGC-3'
Ucp2<br>NM_011671.2	Fwd: 5'-CGAAGCCTACAAGACCATTGC-3'<br>Rev: 5'-ACCAGCTCAGCACAGTTGACA-3'<br>Probe: 5'-CAGAGGCCCCGGATCCCTTCC-3'

SUPPLEMENTARY MATERIALS AND METHODS

Serum and hepatic vitamin A analysis

Serum and tissue retinoid content (both retinol and retinyl palmitate) was analyzed by reverse phase HPLC as previously described [1]. Briefly, tissue (30-50 mg) was homogenized in PBS to create a 15% (w/v) tissue homogenate. Then, tissue homogenate (66.7 µL equal to 10 mg of tissue) or serum (50 µL) were added in the antioxidant mix (containing pyrogallol, butylated hydroxytoluene, ethylenediaminetetraacetic acid and ascorbic acid) and vortexed thoroughly for 1 min. Retinol and retinyl esters were extracted and deproteinized twice with *n*-hexane in the presence of retinol acetate (100 µL, concentration 4 µmol/L) as an internal standard to assess the recovery efficiency after the extraction procedure. Standard curves created from a range of concentrations of retinol and retinyl palmitate were used to determine absolute tissue and serum concentrations of these compounds. Additionally, two negative controls (only containing internal standard) and two positive controls (low and high concentrations of retinol plus internal standard) were included in each series of extractions. Samples were evaporated under N_2 and diluted in 300 µL 100% ultrapure ethanol. Then, 50 µL was injected into HPLC (Waters 2795 Alliance HT Separations Module, Connecticut, USA) for phase separation on a C18 column (Waters Symmetry C18, dimension 150 x 3.0 mm, particle size 5 µm, Waters Corporation, Milford, MA, USA) and measurement (UV-VIS, dual wavelength, UV-4075 Jasco, Tokyo, Japan). Retinoids in samples were identified by exact retention time of known standards in ultraviolet absorption at 325 nm by HPLC. Finally, retinol and retinyl palmitate concentrations were calculated and normalized to final volume or tissue weight.

Quantitative real-time reverse transcription polymerase chain reaction

Quantitative real-time reverse transcription polymerase chain reaction was performed as previously described [2]. Shortly, total RNA was isolated from tissue samples using TRIzol® reagent according to supplier's instructions (ThermoFisher Scientific, Breda, The Netherlands). RNA quality and quantity were determined using a Nanodrop 2000c UV-vis spectrophotometer (Thermo Fisher Scientific). cDNA was synthesized from 2.5 µg of RNA by using random nonamers and M-MLV reverse transcriptase (ThermoFisher Scientific). Taqman primers and probes were designed using Primer Express 3.0.1 (ThermoFisher Scientific) and are shown in **Supplementary Table S1**. All target genes were amplified using the Q-PCR core kit master mix (Eurogentec, Maastricht, The Netherlands) on a 7900HT Fast Real-Time PCR system (ThermoFisher Scientific). SDSV2.4.1 (ThermoFisher Scientific) was used to analyze the data. Expression of genes is presented in $2^{-\text{delta CT}}$ and normalized to *36B4*.

SDS-PAGE and Western Blotting

Protein samples are prepared for Western blot analysis as described previously [3]. Protein concentrations were quantified using the Bio-Rad protein assay (Bio-Rad, Hercules, CA, USA) with bovine serum albumin (BSA) as standard. Equal amounts of protein were separated on Mini-PROTEAN® TGX™ precast 4-15% gradient gels (Bio-Rad) and transferred to nitrocellulose membranes using the Trans-Blot turbo transfer system, (Bio-Rad). Primary antibodies (anti-LRAT, 1:500; # SAB4503589, Sigma-Aldrich), RBP4 (1:2,000; # ab109193, Abcam), ATGL (1:1,000; # 2138, Cell Signaling Technology), PNPLA3 (1:1,000; #PA5-18901, ThermoFisher, scientific), pHSL (1:1,000# 4126, Cell Signaling Technology), HSL (# 4107, Cell Signaling Technology), PEPCK1 (1:500; 10004943, Cayman chem. Abcam), CYP7A1 (H-58): sc-25536, SantaCruz), rabbit-NTCP

(1:1,000; kind gift from Dr. B. Stieger, Zurich, Switzerland [4]), anti-GAPDH, (1:40,000 #CB1001, Calbiochem, Merck-Millipore Amsterdam-Zuidoost, The Netherland) and horseradish peroxidase (HRP)-conjugated secondary antibody (1:2,000; DAKO, Amstelveen, The Netherlands) were used for detection. Proteins were detected using the pierce ECL Western blotting kit (ThermoFisher scientific). Images were captured using the chemidoc XRS system and Image Lab version 3.0, (Bio-Rad). The intensity of bands was quantified using ImageJ version 1.51 (NIH, USA).

Microscopy

Hematoxylin and Eosin (H&E) staining on liver sections (4 µm) was performed on snap-frozen liver sections as previously described [5]. Immunohistochemistry was performed on the paraffin-embedded liver tissue. Briefly, after deparaffinization, antigen retrieval was performed by using microwave irradiation in citrate buffer, pH 6.0 and blocking of endogenous peroxidase with 0.3% H_2O_2 for 30 min. Primary antibodies used were rabbit anti-mouse LRAT (#28075 Takara, Japan). Horse peroxidase-conjugated goat anti-rabbit secondary antibody and rabbit anti-goat tertiary antibodies were used. Slides were stained with the Vector® *NovaRED*™ substrate Kit (# SK-4800, Vector Laboratories, Inc., USA) for 10 min and Haematoxylin was used as a counter nuclear stain for 2 min at room temperature. Finally, slides were dehydrated and mounted with Eukitt® (Sigma-Aldrich). Slides were scanned on a nanozoomer 2.0 digital slide scanner (C9600-12, Hamamatsu Photonics, Hamamatsu, Japan) and analyzed using Aperio ImageScope (version 11.1, Leica Microsystems, Amsterdam, The Netherlands). Autofluorescence analysis was performed on unstained cryostatic liver sections using a Leica CTR 6000 FS fluorescence microscope (Leica Microsystems, Amsterdam, The Netherlands) as previously described

[6,7]. Briefly, cryostat liver sections were illuminated with an excitation filter of 366 nm band-pass interference, and spectra were recorded in the range of 400-680 nm with spectrum acquisition from 0.2 to 3 seconds.

REFERENCES

1. Kim, Y.-K. & Quadro, L. Reverse-phase high-performance liquid chromatography (HPLC) analysis of retinol and retinyl esters in mouse serum and tissues. *Methods Mol. Biol. Clifton NJ* **652**, 263–275 (2010).
2. Blokzijl, H. *et al.* Decreased P-glycoprotein (P-gp/MDR1) expression in inflamed human intestinal epithelium is independent of PXR protein levels. *Inflamm. Bowel Dis.* **13**, 710–720 (2007).
3. Pellicoro, A. *et al.* Human and rat bile acid-CoA:amino acid N-acyltransferase are liver-specific peroxisomal enzymes: implications for intracellular bile salt transport. *Hepatol. Baltim. Md* **45**, 340–348 (2007).
4. Stieger, B. *et al.* In situ localization of the hepatocytic Na+/Taurocholate cotransporting polypeptide in rat liver. *Gastroenterology* **107**, 1781–1787 (1994).
5. Fischer, A. H., Jacobson, K. A., Rose, J. & Zeller, R. Hematoxylin and eosin staining of tissue and cell sections. *CSH Protoc.* **2008**, pdb.prot4986 (2008).
6. Croce, A. C. *et al.* Human liver autofluorescence: an intrinsic tissue parameter discriminating normal and diseased conditions. *Lasers Surg. Med.* **42**, 371–378 (2010).
7. Croce, A. C. *et al.* Liver autofluorescence properties in animal model under altered nutritional conditions. *Photochem. Photobiol. Sci. Off. J. Eur. Photochem. Assoc. Eur. Soc. Photobiol.* **7**, 1046–1053 (2008).
